# Multidisciplinarity and Trandisciplinarity in the Diagnosis and Treatment of Pediatric Gastrointestinal Diseases

**DOI:** 10.3390/diagnostics14080852

**Published:** 2024-04-20

**Authors:** Cristina Oana Mărginean

**Affiliations:** Department of Pediatrics I, University of Medicine, Pharmacy, Sciences and Technology George Emil Palade of Târgu Mureș, 540136 Târgu Mureș, Romania; marginean.oana@gmail.com

It is an honor and a privilege to have helped bring this Special Issue titled “Multidisciplinarity and Trandisciplinarity in the Diagnosis and Treatment of Pediatric Gastrointestinal Diseases” to you. 

The specialty of pediatric gastroenterology has experienced accelerated development in recent decades through its development as a subspecialty and later a specialty from its “mother” discipline, pediatrics. This occurred due to its tendency, in the recent years, to be more and more specialized in a smaller area and because of improvements in diagnosis and therapeutic techniques and skills associated with this discipline, such as ultrasound, endoscopy, echo-endoscopy, or endoscopic retrograde cholangiopancreatography [1]. Thus, pediatric gastro-entero-intestinal diseases are problems that require diagnosis and short- and long-term management for rare disorders and complex cases of more common disorders that affect the gastrointestinal tract; moreover, these diseases frequently need a multidisciplinary team in order to provide adequate management [2]. 

The purpose of this Special Issue is to allow researchers from around the world to report on the new insights into pediatric gastrointestinal diseases. Thus, we aim to include papers related to both the diagnosis and management of a wide spectrum of pediatric gastrointestinal diseases, such as celiac disease, acute and chronic diarrhea, inflammatory bowel disease, food allergies, nutritional disorders, gastritis, and other functional gastrointestinal disorders. Assessing and reporting novel information regarding different gastrointestinal disorders is highly important as it is a major concern of pediatricians in clinical practice. It is a well-documented fact that gastrointestinal diseases represent the most important cause of morbidity in pediatric patients. The proper diagnosis and management of these pathologies independently of their organic or functional etiology will create healthier adult populations, decreasing the costs related to health services and improving the care that might have to be individualized for each patient.

Therefore, an important topic in this area is pediatric gastroesophageal reflux disease (GERD), whose manifestations can be divided into esophageal and extraesophageal syndromes, with the latter including respiratory tract changes such as reflux cough syndrome, reflux laryngitis syndrome, asthma, wheezing, reflux dental erosion syndrome, pharyngitis, sinusitis, as well as irritability, failure to thrive, anemia, feeding refusal dystonic neck posturing (Sandifer syndrome), and other neurological symptoms that need an experienced gastroenterologist and a multidisciplinary team to carry out correct management [3].

Another challenge in pediatric gastrointestinal diseases is offered by eosinophilic esophagitis (EoE), an immune-mediated disease that produces esophageal dysfunction secondary to an increase in the eosinophil count, in the absence of other diseases, and needs histopathological confirmation (histology scoring system) [4,5]. EoE needs a correct diagnosis and an appropriate therapy to be selected to prevent complications [4], such as esophageal stenosis or perforation [6]. The treatment consists of proton pump inhibitors (PPIs), corticosteroids, and a dietary regimen (the elimination of foods) [4].

Of course, the most complex field that constantly transcends boundaries is represented by the *Helicobacter pylori* (*H. pylori*) infection—with high incidence in children, especially in teenagers—which is responsible for gastrointestinal complications (chronic active gastroenteritis, gastric and duodenal ulcers, gastric cancer), but which has also been incriminated as a key player in the development of extra-digestive conditions, including neurological, cardiac, metabolic, hematologic (immune thrombocytopenic purpura, anemia), ocular, and dermatological pathologies. The discovery of the modulatory effect of *H. pylori* upon the gut–brain axis and the gastric microenvironment suggested possible systemic effects of the infection [7,8]. Neurological manifestations include cognitive impairment, migraine, or Alzheimer’s disease, which have been extensively studied, but without a clear pathophysiology of the process [9]. Nevertheless, *H. pylori* has been associated with a variety of innate as well as acquired autoimmune disorders [10].

Cystic fibrosis (CF), an autosomal recessive monogenic disease, is a multiorgan disorder affecting the respiratory tract, exocrine pancreas, intestine, hepatobiliary system, sweat glands, and myeloid cells [11,12]. It has a chronic, progressive, and potentially fatal course of development. The gene responsible for cystic fibrosis is the transmembrane conductance regulator (CFTR) protein, with multiple well-known and studied mutations, out of which F508del is the most frequent [11]. Although the respiratory symptoms dominate the clinical picture in CF, the gastrointestinal manifestations should be recognized and treated promptly as they can lead to exocrine pancreas involvement, distal intestinal obstruction syndrome (DIOS), and small intestinal bacterial overgrowth (SIBO), whose management is very important for the quality of life in patients with CF [11].

Some digestive chronic diseases, such as inflammatory bowel disease (IBD), can have an unpredictive evolution, and even become debilitating. The constant fatigability, short stature, growth delay, and delayed puberty that can accompany these conditions can impair an individual’s mental and emotional wellbeing and place them at a higher risk for developing psychiatric conditions. Therefore, a quick diagnosis and a good management of these diseases can prevent psychiatric distress, which can later ensure prophylaxis of psychiatric disorders, later in adult life [13].

Several viruses with traditional tropism for the gastrointestinal mucosa have been regarded as major factors of gastroenteritis-associated morbidity and mortality, especially at a young age. Still, viral infections that usually cause respiratory tract infections can also determine digestive manifestations. Although the SARS-CoV-2 infection frequently produces respiratory symptoms, its Omicron variant poses 1.5-fold higher odds of determining loss of appetite in the infected individual and 1.6-fold more frequent digestive symptoms in infants between 7 and 9 months of age. Moreover, its frequent association with hepatic cytolysis has also been reported [14].

Through this Special Issue, we aimed to emphasize that multidisciplinarity and trandisciplinarity in pediatric gastrointestinal diseases are very useful for providing correct management of children affected by these diseases. The topics addressed within this collection of articles have covered the vast majority of pediatric digestive pathology and have included research articles, reviews, and clinical case reports. The articles were written by experts in their fields, with high expertise in clinical management of the disorders that involve crossing the borders between specialties. The published articles aided in providing a better understanding of the pathogenesis, outlined management controversies of pediatric gastrointestinal disorders, and enriched literature data. All these articles reflect the progress in the field and the future steps in pediatric gastroenterology should be undertaken. Editing this Special Issue was a great, delightful experience for me—a learning opportunity—and I hope for that it will prove similar for its audience.

## References

[1] ESPGHAN Training Syllabus 2019|ESPGHAN. http://www.espghan.org/knowledge-center/education/ESPGHAN_Training_Syllabus_2019.

[2] Paediatric Gastroenterology, Hepatology and Nutrition—Sub-Specialty. https://www.gmc-uk.org/-/media/documents/appendix-4i---pghan_pdf-86370658.pdf.

[3] Rosen R., Vandenplas Y., Singendonk M., Cabana M., DiLorenzo C., Gottrand F., Gupta S., Langendam M., Staiano A., Thapar N. (2018). Pediatric Gastroesophageal Reflux Clinical Practice Guidelines: Joint Recommendations of the North American Society for Pediatric Gastroenterology, Hepatology, and Nutrition and the European Society for Pediatric Gastroenterology, Hepatology, and Nutrition. J. Pediatr. Gastroenterol. Nutr..

[4] Dhar A., Haboubi H.N., Attwood S.E., Auth M.K.H., Dunn J.M., Sweis R., Morris D., Epstein J., Novelli M.R., Hunter H. (2022). British Society of Gastroenterology (BSG) and British Society of Paediatric Gastroenterology, Hepatology and Nutrition (BSPGHAN) Joint Consensus Guidelines on the Diagnosis and Management of Eosinophilic Oesophagitis in Children and Adults. Gut.

[5] Collins M.H., Martin L.J., Alexander E.S., Boyd J.T., Sheridan R., He H., Pentiuk S., Putnam P.E., Abonia J.P., Mukkada V.A. (2017). Newly Developed and Validated Eosinophilic Esophagitis Histology Scoring System and Evidence That It Outperforms Peak Eosinophil Count for Disease Diagnosis and Monitoring. Dis. Esophagus.

[6] Straumann A., Bussmann C., Zuber M., Vannini S., Simon H.-U., Schoepfer A. (2008). Eosinophilic Esophagitis: Analysis of Food Impaction and Perforation in 251 Adolescent and Adult Patients. Clin. Gastroenterol. Hepatol..

[7] Baj J., Forma A., Sitarz M., Portincasa P., Garruti G., Krasowska D., Maciejewski R. (2020). *Helicobacter Pylori* Virulence Factors-Mechanisms of Bacterial Pathogenicity in the Gastric Microenvironment. Cells.

[8] Mayer E.A., Tillisch K., Bradesi S. (2006). Review Article: Modulation of the Brain-Gut Axis as a Therapeutic Approach in Gastrointestinal Disease. Aliment. Pharmacol. Ther..

[9] Pellicano R., Ribaldone D.G., Fagoonee S., Astegiano M., Saracco G.M., Mégraud F. (2016). A 2016 Panorama of Helicobacter Pylori Infection: Key Messages for Clinicians. Panminerva Med..

[10] Wang L., Cao Z.-M., Zhang L.-L., Dai X., Liu Z., Zeng Y., Li X.-Y., Wu Q.-J., Lv W. (2022). Helicobacter Pylori and Autoimmune Diseases: Involving Multiple Systems. Front. Immunol..

[11] Fiorotto R., Strazzabosco M. (2019). Pathophysiology of Cystic Fibrosis Liver Disease: A Channelopathy Leading to Alterations in Innate Immunity and in Microbiota. Cell Mol. Gastroenterol. Hepatol..

[12] Betapudi B., Aleem A., Kothadia J.P. (2024). Cystic Fibrosis and Liver Disease. StatPearls.

[13] Sălcudean A., Nan A.G., Bodo C.R., Cosma M.C., Strete E.G., Lica M.M. (2023). Association between Childhood Onset Inflammatory Bowel Disease and Psychiatric Comorbidities in Adulthood. Diagnostics.

[14] Drăgănescu A.C., Miron V.D., Săndulescu O., Bilaşco A., Streinu-Cercel A., Sandu R.G., Marinescu A., Gunșahin D., Hoffmann K.I., Horobeț D.Ș. (2023). Omicron in Infants-Respiratory or Digestive Disease?. Diagnostics.

